# Biological and Physicochemical Characterization of Biodegradable Aliphatic Polyesters with Copper Deposited by Magnetron Sputtering

**DOI:** 10.3390/ma19010057

**Published:** 2025-12-23

**Authors:** Malgorzata Latos-Brozio, Aleksandra Drzazga, Anna Masek, Zdzisława Mrozińska, Marcin H. Kudzin

**Affiliations:** 1 Institute of Polymer and Dye Technology, Faculty of Chemistry, Lodz University of Technology, Stefanowskiego 16, 90-537 Lodz, Poland; aleksandra.drzazga@p.lodz.pl (A.D.); anna.masek@p.lodz.pl (A.M.); 2 Łukasiewicz Research Network—Łódź Institute of Technology, Marii Sklodowskiej-Curie 19/27, 90-570 Lodz, Poland; zdzislawa.mrozinska@lit.lukasiewicz.gov.pl

**Keywords:** aging, antimicrobial activity, biodegradable materials, blood coagulation, copper magnetron sputtering, polycaprolactone (PCL), polylactide (PLA)

## Abstract

Biodegradable polymer materials, which reduce the problem of waste and are often produced from renewable raw materials, contribute to sustainable development. The imparting of antimicrobial properties to biodegradable materials represents a significant advantage in a variety of potential applications, including the domain of packaging materials and medical applications. In this study, biodegradable polymer compositions, including polylactide (PLA) and polycaprolactone (PCL), were prepared with copper, which was applied to the polymers using a magnetron sputtering technique. PLA and PCL were selected as representatives of biodegradable polymers of natural and synthetic origin. Copper was used as an alternative to other more expensive metals with antimicrobial properties. The microbiological properties of the samples were examined, the ultraviolet protection factor (UPF) was determined, and the influence of controlled thermo-oxidative and weathering aging on the surface properties of the materials (color, wettability, surface energy, UV-Vis spectra) was analyzed. The UPF values for the PLA and PCL samples containing copper were UPF > 50, so the materials provided excellent UV protection. Thermo-oxidative aging of PCL and weathering aging of PLA influenced the change in color and surface properties (wettability and surface energy) of the composition, resulting from the oxidation of the copper layer deposited on the polymers. Biological evaluation included measurements of prothrombin time (PT) and activated partial thromboplastin time (aPTT) to assess how the synthesized materials influence the intrinsic and extrinsic pathways of blood coagulation, reflecting their potential biomedical relevance. Furthermore, the antimicrobial performance of the obtained samples was examined against representative bacterial strains—*Staphylococcus aureus* (Gram-positive) and *Escherichia coli* (Gram-negative)—to verify their ability to inhibit microbial growth and ensure their suitability for use in infection-prone environments.

## 1. Introduction

Polymer products made from traditional plastics have many inherent problems that exacerbate environmental degradation and pose a serious challenge to sustainability [1]. Consequently, the concept of ‘sustainable’ polymers has emerged, attracting significant attention due to their ability to resolve numerous concerns pertaining to environmental impact. The term “sustainable polymers” (also called “bioplastics”) covers both biodegradable polymers and bio-based polymers, wholly or partially made from renewable raw materials [2].

Polylactide (PLA) is one of the most popular and widely used bioplastics [3,4,5,6], and its high commercial availability is accompanied by a competitive price [2,7]. Polylactide is a thermoplastic polyester of natural origin, and the PLA monomer, lactic acid, is obtained by fermenting carbohydrates [8] derived from materials such as starch [9], food waste [10], and biomass [11]. Polylactide is one of the major biodegradable polymers derived from renewable sources due to its properties such as flexibility, biocompatibility, excellent mechanical properties, good processability, and optical transparency [12,13,14]. Thanks to its properties, PLA is used in many different fields, such as pharmaceuticals, orthopaedics, cosmetics, food packaging, textiles, electronics, and transportation [15,16]. The biodegradation of polylactide is a process that occurs through a series of chemical and microbiological reactions, resulting in the breakdown of the polymer into smaller components. PLA degradation is primarily attributable to the disintegration of ester bonds, a consequence of both alkaline and enzymatic hydrolysis [12]. Composting PLA allows for the production of CO_2_ and H_2_O, but the process requires temperatures close to the glass transition temperature of the polymer (60 °C) and high relative humidity. It has been demonstrated that the initial absorption of carbon dioxide (CO_2_) during the production of PLA serves to counterbalance the resulting emissions. In such conditions, the degradation time can be as short as 30 days [17].

In addition to biodegradable polymers derived from renewable raw materials, there are also biodegradable polymers derived from petroleum-based materials, such as polycaprolactone (PCL). Polycaprolactone is an aliphatic polyester classed as biocompatible and biodegradable in various environmental conditions, including compost, soil and aquatic environments, mainly due to the activity of microbial enzymes. This property differentiates PCL from numerous conventional petroleum-based polymers, which remain in the environment for a long time. PCL has attracted significant attention in the field of biomedical applications due to its slow degradation rate and excellent biocompatibility. These characteristics render it suitable for use in a variety of applications, including biodegradable implants, tissue engineering as well as drug delivery systems [18]. The primary PCL monomer, ε-caprolactone, is commonly derived from fossil sources, irrespective of the polymer’s biodegradability. However, given the environmental concerns associated with the use of petrochemicals, there have been reports in the literature of studies on new, renewable methods for synthesising ε-caprolactone [19].

The addition of new functionalities to biodegradable polymers, including antimicrobial properties, has been the subject of numerous publications [20,21,22,23,24]. Due to their high antibacterial efficacy, simplicity, and low technological preparation costs, polymer-inorganic hybrids are becoming more and more important [25,26]. Copper is gaining increasing popularity among various antibacterial agents [27,28,29] because of its properties such as chemical stability, developed surface area, and prolonged antibacterial activity, as well as the good abrasion resistance of copper layers [25,30,31]. Moreover, compared to other antibacterial materials based on precious metals such as silver or gold, copper is significantly less expensive. Copper-based materials are more readily available, and copper nanoparticles can be synthesized in an environmentally friendly manner with good biological compatibility. Copper nanoparticles can dissolve, releasing free ions more quickly than precious metal nanoparticles, which translates into copper ions being more effective in generating reactive oxygen species (ROS), exerting antimicrobial activity, and undergoing chemical catalysis [32]. Research on other potentially antibacterial nanoparticles based on ZnO or Pd is not as extensive as that on Ag, Au, or Cu, and future efforts may be undertaken to elucidate the detailed mechanism of action of these nanoparticles [32].

Copper is an essential and fundamental element of physiological processes in the body. However, its active nature can cause toxicity to mammalian cells or lead to long-term toxicity in humans after its accumulation in the body. Unlike noble metals used in antibacterial applications, copper oxidizes more readily and tends to dissolve as ions. This property gives copper its broad antibacterial applications, but the resulting toxicity is also a significant factor, which must be considered when designing copper-containing materials [32]. Copper is an essential trace element, its biological effects are strongly dose-dependent, and toxicity is mainly associated with excessive systemic exposure rather than surface-confined copper species [33,34,35]. In antimicrobial copper-based coatings, the antibacterial activity is commonly attributed to a combination of contact-mediated effects and the local presence of copper species at the material interface, rather than uncontrolled bulk release [36,37].

The antibacterial activity exhibited by the metallic copper surface is attributable to two complementary mechanisms: surface-surface interaction of copper and bacteria (contact killing) and/or surface oxidation of copper with subsequent release of antibacterial copper (II) ions [37,38,39,40,41,42]. Polymer–Cu–nanoparticle systems have been prepared using a wide range of chemical, biological, and physical synthesis methods [43,44,45,46], including the magnetic sputtering method [47,48]. The technique of metal deposition using magnetron sputtering has many advantages, including that the method is simple and environmentally friendly, and allows the required amount of metal to be deposited depending on the application time [26]. According to literature reports, biodegradable polymer systems coated with copper have been described. PLA–Cu^o^ material was produced by spraying copper ions onto polylactide nonwovens using the melt blown method [25,26]. Composite fibres with antimicrobial properties made of polylactide-copper alginate (PLA–ALG Cu^2+^) are also known [49]. D. J. da Silva and co-authors used radio frequency magnetron sputtering (RF-MS) to produce antimicrobial copper coatings on the surface of PLA. According to the authors, a PLA coating with copper deposited in a short time (5–20 s) was sufficient to guarantee bactericidal activity against *Escherichia coli* and *Bacillus subtilis*, as well as antiviral activity against severe acute respiratory syndrome coronavirus 2 (SARS-CoV-2) [50]. A. Munoz-Escobar et al. [51] proposed polycaprolactone (PCL) film with copper oxide nanoparticles (CuONPs), which showed significant antibacterial activity against Gram-positive bacteria, as a wound dressing.

There is a lack of data in the literature on the impact of ageing of copper coatings applied to biodegradable materials under the influence of various external factors, such as oxidation, elevated temperatures or weathering conditions. Data on the durability of metallic coatings during ageing processes and the exploitation of polymer materials need to be supplemented.

The aim of this study is to evaluate the physicochemical and biological properties of biodegradable polymer composites based on polylactide (PLA) and polycaprolactone (PCL) containing copper deposited by magnetron sputtering. Magnetron sputtering was chosen over wet chemical methods because it avoids the use of toxic solvents, which aligns with the principles of green chemistry. Magnetron sputtering offers a solvent-free, reproducible, and tunable deposition strategy, enabling stable antimicrobial surface functionalization without introducing additional organic extractables into the polymer matrix [52]. The research focuses on assessing the antimicrobial potential of the obtained materials and their stability under accelerated ageing conditions. In addition, the effect of controlled PLA weathering ageing and PCL thermo-oxidative ageing on surface characteristics was analysed, including wettability, surface energy, colour changes, and UV-Vis spectral variations. The ultraviolet protection factor (UPF) was also determined to assess the protective performance of the copper layer. Biological characterization encompassed prothrombin time (PT) and activated partial thromboplastin time (aPTT) measurements to evaluate the influence of the composites on blood coagulation. The combined evaluation of surface and biological responses of copper-modified biodegradable polymers provides insights into their potential applications in biomedical and packaging materials, as well as in estimating their durability and degradation behaviour over time.

## 2. Materials and Methods

Preparation of PLA and PCL samples: Polylactide granulate type Ingeo 4043D (Nature Works, Minnetonka, MN, USA) or polycaprolactone granulate (6-caprolactone polymer, average Mn 80,000, Aldrich, Germany) were used for the tests. Disc-shaped samples were prepared by pressing the plasticized polymer. The extruder was used to plasticize the polymer mass, which was removed directly from the last heating zone of the extruder into molds. The granules were extruded an Axon ab Plastic Machinery S-265 50 NYVANG extruder (Stockholm, Sweden). The temperatures of the individual extruder zones for PLA and PCL were as follows: PLA—zone I 195 °C, zone II 245 °C, zone III 260 °C, and screw speed 130 rpm; PCL—zone I 70 °C, zone II 80 °C, zone III 90 °C, and screw speed 130 rpm. Discs of a specific size were formed by pressing the polymer mass in molds (pressure approx. 0.5–1 MPa, time 3 min). Samples in the shape of discs with a diameter of 3 cm and a thickness of 0.4 cm were obtained. Four discs were produced from each polymer from the same extrusion batch.

PLA and PCL discs were modified using a DC magnetron sputtering system from P.P.H. Jolex s. c. (Częstochowa, Poland). A copper target (Testbourne Ltd., Basingstoke, UK) with a purity of 99.99% was used. The coatings were deposited in an argon atmosphere (3%). The target–sample distance was 15 cm. Due to the large size of the magnetron used, all samples were sputtered simultaneously. This means that all samples were placed in the magnetron chamber and sputtered on both sides for 10 min each (total sputtering time 20 min). Half of the samples were then removed, and the remaining samples were re-sputtered for 10 min on each side (total sputtering time 20 min + 20 min = 40 min). The designations 20 and 40 denote the sample sputtering time. The base pressure was typically about 10^−6^ mbar. The following parameters were used for modification:

Conditions 1: Power 0.5 kW, circulating power 0.25 kW, operating pressure 2.3 × 10^−3^ mbar, sputtering time for 1 side of the samples 10 min, total sputtering time for both sides of the samples 20 min.

Conditions 2: Power 0.5 kW, circulating power 0.25 kW, working pressure 2.3 × 10^−3^ mbar, sputtering time for 1 side of the samples 20 min, total sputtering time for both sides of the samples 40 min.

Table 1 presents the samples and the conditions under which they were produced.

Determination of copper content in PLA and PCL samples: The copper content in PLA and PCL samples was determined using a Magnum II single-module microwave mineraliser from Ertec (Wrocław, Poland) and a Thermo Scientific Thermo Solar M6 atomic absorption spectrometer (LabWrench, Midland, ON, Canada) equipped with a 100 mm titanium burner, coded lamps with a single-component cavity cathode, background correction: D2 deuterium lamp. The detection limit and quantification limit of the method were: LOD = 0.25 mg/L, a LOQ = 0.23 mg/L; LOD = 10.59 mg/kg, a LOQ = 11.66 mg/kg.

Before determining the copper content in the samples, the materials were mineralized. Samples weighing 0.1 g were mineralized in a mixture of deionized water, H_2_O_2_ (30%), and 65% nitric acid in a 2:3:5 ratio. The mineralization conditions were as follows:

Cycle 1: heating time 15 min, pressure 20–30 bar, temperature 200–250 °C
Cycle 2: heating time 15 min, pressure 26–38 bar, temperature 200–250 °C
Cycle 2: heating time 15 min, pressure 30–44 bar, temperature 200–250 °C

The total copper content in PLA and PCL samples [mg/kg; ppm] was calculated according to Equation (1)

(1) $$M=\frac{Ci\times V}{mi}$$

where

*Ci*—metal concentration in the analyzed solution [mg/L];
*mi*—mass of the mineralized sample PLA or PCL [g];
*V*—volume of the sample solution [mL].

UPF Ultraviolet Protection Factor (UPF): The measurements were performed in accordance with EN 13758-1:2002 [53]. The tests were performed using a Jasco V-550 spectrophotometer (Tokyo, Japan) in the range of 290–800 nm. Four measurements were made on both sides of the samples and the results were averaged. The UPF index was calculated using the following Formula (2):

(2) $$\mathrm{UPF}=\frac{\Sigma_{\lambda =290}^{\lambda =400}E(\lambda )\varepsilon (\lambda )\Delta (\lambda )}{\Sigma_{\lambda =290}^{\lambda =400}E(\lambda )T(\lambda )\varepsilon (\lambda )\Delta (\lambda )}$$

where

E(λ)—the solar irradiance
ε(λ)—the erythema action spectrum, measure of the harmfulness of UV radiation for human skin
Δ(λ)—the wavelength interval of the measurements
T(λ)—the spectral transmittance at wavelength λ

The UPF value of the samples was determined as the arithmetic mean of the UPF values for each sample, reduced by a statistical value depending on the number of measurements taken, with a confidence interval of 95%.

Colour of PLA and PCL compositions: The colour testing was conducted using a spectrophotometer CM-3600d (Konica Minolta Sensing, Osaka, Japan). The measurements were performed on unaged samples in order to ascertain the effect of copper magnetron sputtering on colour change. In addition, the colour change in both PLA following weathering ageing and PCL following thermo-oxidative ageing was determined. Three measurements were made on both sides of the samples and the results were averaged. Measurement error is given as standard deviation. The result of the examination is the colour as described in the CIE-Lab space and the colour in a system of three coordinates: In the context of colour theory, L, A and B are three distinct parameters that are of significance in the analysis of lightness. The parameter L, denoted as the lightness parameter, assumes a maximum value of 100, which is represented by a perfectly reflecting diffuser. Conversely, the minimum value of 0 is represented by the colour black. The parameters A and B are defined by the axes of red-green and yellow-blue, respectively. The a and b axes are not subject to specific numerical limits. The change in colour, dE × ab, was computed according to the following Equation (3):

(3) $$dE\times ab=\sqrt{({∆a}^{2})+\left({∆b}^{2}\right)+\left({∆L}^{2}\right)}$$

UV-Vis analyses for PLA and PCL: The spectra of PLA and PCL samples with copper deposited by magnetron sputtering samples at wave-lengths of 190 to 1100 nm were recorded utilizing a UV-Vis spectrophotometer (Evolution 220, Thermo Fisher Scientific, Waltham, MA, USA).

Surface free energy (SEP): The obtained samples based on PLA and PCL were characterized by surface free energy. Initially, the contact angles of each material were measured by utilizing goniometer OCA 15EC (DataPhysics Instruments GmbH from Filderstadt, Germany) with SCA 20 module software. Calculations of SFE were determined by OWRK method (Owens-Wendt-Rabel-Kealble), which is based on measurements of contact angles using polar and non-polar liquids (in this work were used distilled water and diiodomethane), thanks to which dispersive and polar components can be obtained and calculated to surface energy, as shown in Equation (4) [54]:

(4) $$E_{T}=E_{P}+E_{D}$$

where $E_{T}$ is free surface energy (total value), consisting of polar ($E_{P}$) and dispersive ($E_{D}$) components, presented in units [mJ/m^2^]. Calculation of polar and non-polar constituents, referring to OWRK method were presented by Equations (5) and (6) [55,56]:

(5) $${\left(E_{P}\right)}^{0.5}=\frac{E_{w}\cdot \left({cos\;\theta }_{w}+1\right)-2\cdot \sqrt{E_{w}^{D}\cdot E_{D}}}{2\cdot \sqrt{E_{w}^{P}}}\;$$

(6) $${\left(E_{D}\right)}^{0.5}=\frac{E_{d}\cdot \left({cos\;\theta }_{d}+1\right)-\sqrt{\frac{E_{d}^{P}}{E_{w}^{P}}}\cdot E_{w}\cdot \left({cos\;\theta }_{w}+1\right)}{2\cdot \left(\sqrt{E_{d}^{D}}-\sqrt{E_{d}^{P}\cdot \frac{E_{w}^{D}}{E_{w}^{P}}}\right)}\;$$

where $E_{w}$ is water surface energy (72.6 mJ/m^2^), $E_{w}^{D}$ and $E_{w}^{P}$ are dispersive (21.6 mJ/m^2^) and polar (51.0 mJ/m^2^) components. $E_{d}$ is diiodomethane surface energy (50.8 mJ/m^2^) and $E_{d}^{D}$, $E_{d}^{P}$ are its dispersive (48.5 mJ/m^2^) and polar (2.3 mJ/m^2^) components.

For each material, three measurements of contact angles were taken, using previously mentioned two liquids: distilled water and diiodomethane. Measurements were taken on both sides of the samples and the results were averaged. The value errors of measured contact angles and surface energy parameters were calculated using the *t*-test statistic (Hypothesis Testing, One Sample *t*-test) with operating OriginLab program.

Ageing of samples: The polymer compositions were aged for 7 days (168 h). The PLA was subjected to ageing in an Atlas Weather Ometre Ci 4000 climate chamber (Atlas Material Testing Technology, Mt. Prospect, IL, USA), and the test consisted of two alternating segments with the following parameters: daytime panel, i.e., 240 min, radiation intensity 0.7 W/m^2^, humidity 60%, and night-time panel, i.e., 120 min, humidity 50%, no UV radiation. PCL was subjected to thermo-oxidative ageing at 50 °C in a heating chamber (Binder, Germany) with forced convection. The PLA and PCL were subjected to different ageing chambers due to their varied resistance to the ageing conditions, including PCL’s low thermal resistance.

Antimicrobial Activity: The antimicrobial activity of the samples coated with copper via magnetron sputtering was evaluated in accordance with the EN ISO 20645:2006 standard [57]. The tests were performed using the agar diffusion plate method on Mueller–Hinton agar to assess antibacterial performance against representative bacterial strains. Two model microorganisms were selected: *Staphylococcus aureus* (ATCC 6538) as a Gram-positive strain and *Escherichia coli* (ATCC 25922) as a Gram-negative strain. Sterilized Mueller–Hinton agar was poured into Petri dishes and inoculated with bacterial broth cultures prepared under aseptic conditions. After solidification, circular polymer samples were gently placed on the inoculated agar surface. The plates were then incubated for 24 h at 37 °C under static conditions. Following incubation, the growth of bacteria in direct contact with the samples and in the surrounding zones was visually examined to determine the antimicrobial response according to EN ISO 20645:2006 evaluation criteria. All tests were performed in duplicate to ensure reproducibility.

Measurement of Activated Partial Thromboplastin Time (aPTT) and Prothrombin Time (PT): Human plasma in frozen and lyophilized form was reconstituted with deionized water immediately before analysis. For each measurement, 1 mg of the reconstituted material was mixed with 200 µL of plasma and subsequently centrifuged. The resulting suspension was incubated for 15 min at 37 °C to ensure thermal equilibration prior to testing. The activated partial thromboplastin time (aPTT) was determined using a Dia-PTT diagnostic reagent containing kaolin, cephalin, and 0.025 M calcium chloride (CaCl_2_). Measurements were carried out on a K-3002 OPTIC coagulometer. During each test, 50 µL of plasma and 50 µL of the Dia-PTT reagent were introduced into the instrument’s thermostatic chamber maintained at 37 °C. After a 3 min preincubation period, 50 µL of the CaCl_2_ solution was added to trigger the coagulation reaction, and clotting time was automatically recorded. The prothrombin time (PT) was measured under analogous conditions. In this case, 100 µL of plasma was preincubated for 2 min at 37 °C, followed by the addition of 100 µL of a thromboplastin suspension (Dia-PTT reagent). The reagent consisted of rabbit brain–derived thromboplastin, calcium ions, and a preservative. The suspension was thoroughly homogenized before each assay to ensure reproducibility. All measurements were performed in duplicate, and the obtained coagulation times were expressed in seconds. Measurement error is given as standard deviation.

## 3. Results and Discussion

The study commenced with the determination of the content of magnetron-sputtered copper on PLA and PCL samples (Table 2). The PLA compositions contained 0.68 g/kg and 4.00 g/kg of copper, respectively, in the 20- and 40-min magnetron-sputtered materials. As the magnetron copper deposition time increased, the metal content in the samples was higher. For PCL samples, regardless of the magnetron metal deposition time, the copper content in the samples was similar and ranged from 3.55 to 4.43 g/kg. Each measurement was performed in triplicate, and the reported values represent the mean of three independent determinations.

Table 3 summarises the results of the ultraviolet protection factor (UPF) and parameters related to the determination of UPF indices, such as the amount of UVA and UVB radiation penetrating PLA and PCL materials and the average transmittance (T%) measured in the range of λ = 290–400 nm. The reference polymer materials had the following UPF factors: PLA 4.84 and PCL 7.96. Due to its transparent nature, polylactide had a lower UPF factor, higher UVA and UVB radiation permeability, and higher average transmittance than the milky-white polycaprolactone polymer. After magnetron copper deposition, the UPF coefficients for all samples were >50, which means that the materials provide excellent protection against ultraviolet radiation, blocking over 97.5% of sunlight. Meanwhile, the transmittance of UVA and UVB radiation, as well as the average T were 0.01–0.02% for samples coated with metal. The copper layer could act as a physical barrier limiting the penetration of UVA and UVB radiation through polyester samples and provided UPF factors above 50.

The effect of controlled ageing on the physicochemical properties of PLA and PCL compositions containing magnetron-sputtered copper was analysed. The colour change in the materials was examined, UV-Vis spectra of the samples were measured, and the contact angles and surface energy of the compositions were determined before and after weathering ageing for PLA and thermo-oxidative ageing for PCL. The different thermal properties of PLA and PCL polymers pose a limitation, and direct comparison of copper layer degradation between the two materials during controlled aging is difficult because the PLA samples underwent UV photodegradation, while the PCL samples did not.

**Figure 1:**
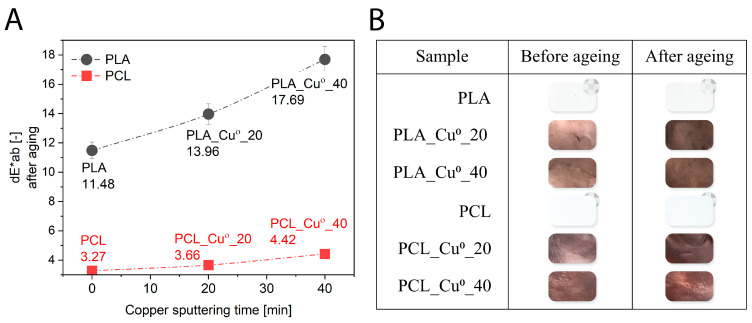
Colour change coefficients dE × ab (**A**) and visual colour change (**B**) of PLA_Cu^o^ and PCL_Cu^o^ samples after ageing.

As illustrated in Figure 1A, the colour change coefficients (dE × ab) of the samples were measured after undergoing ageing processes within ageing chambers. Photographic documentation (Figure 1B) was also conducted to illustrate the visual colour changes observed in the materials. As a result of weathering ageing, the transparent reference PLA sample clearly changed colour to milky, which corresponded to a dE × ab = 11.48 (-) value. According to statistical analysis of colour change coefficients, dE × ab values above 5 units indicate that the colours of the samples are perceived as completely different. The noticeable change in colour (transparency) of PLA was associated with a change in the crystalline phase content in the reference sample due to the action of adverse degrading factors (UV radiation, elevated temperature, humidity) during ageing. The increase in PLA crystallinity after exposure to atmospheric conditions has been described in the literature [58]. The breakdown of the polymer chain, which occurs during ageing, promotes rearrangement in the amorphous zones of the polymer, followed by recrystallisation, increasing crystallinity [58], which may affect the optical properties of the PLA sample. For the reference PCL sample aged by thermal-oxidation, milder degradation factors, mainly a temperature of 50 °C, may have contributed to a smaller change in the colour of the polymer matrix (dE × ab = 3.27 (-)). In the range of 2 < dE × ab < 3.5, the colour difference is discernible to the average observer. The colour change coefficients of the reference PLA and PCL polymers correspond to the UPF indices. Polylactide with a lower ultraviolet protection factor and allowing more UVA and UVB radiation to pass through was more susceptible to colour change associated with polymer degradation processes.

The process of ageing of samples of polylactide under climate conditions and of poly(caprolactone) under thermo-oxidative conditions resulted in a significant alteration in their colour. Under the influence of degrading factors, the copper coating changed colour from a metallic orange shade to a darker brown. The characteristic darkening of colour is attributable to the fact that copper, when exposed to the environment or in a laboratory setting, forms a brown-black layer of cuprite (Cu_2_O) [59,60,61]. In general, oxide growth can occur when copper surfaces come into contact with an oxygen-rich environment. It has been assumed that the sequence of reactions leading to the oxidation of a clean copper surface is oxygen chemisorption, nucleation and growth of surface oxide, and growth of bulk oxide. The oxidation process can result in the formation of two most common forms of oxide: cuprite (or copper oxide, Cu_2_O), the main oxide at low temperatures and low pressure, and tenorite (or copper oxide, CuO), which dominates at high temperatures and high pressure. In ambient conditions the primary oxide is cuprite [62,63]. The oxide layer consists mainly of Cu_2_O, and CuO begins to form at high temperatures and with long oxidation times. The formation of CuO was observed in quartz crystal microbalance (QCM) measurements at temperatures of 90 °C and 100 °C, when the oxidation time was hundreds of hours [63].

PLA and PCL materials coated with copper demonstrated analogous behaviour; as the duration of metal coating increased, the change in colour of the composition after ageing was greater. On the surface of PLA_Cu^o^_40 and PCL_Cu^o^_40 samples containing more copper, a greater amount of cuprite could have formed after weathering and thermal oxidation ageing, hence the colour change after degradation was more pronounced.

**Figure 2:**
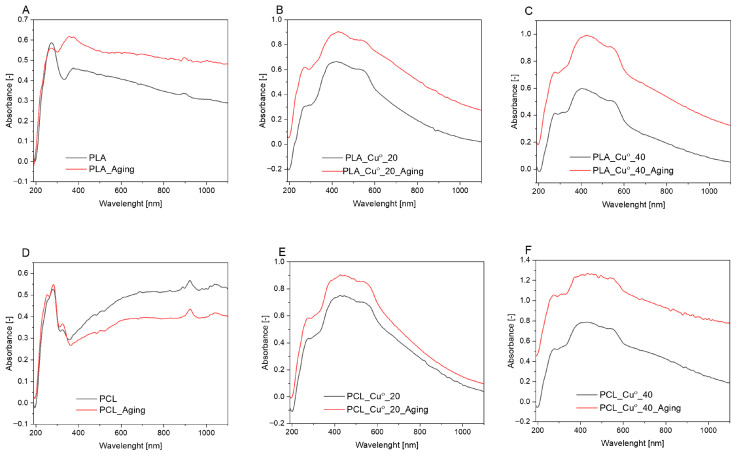
UV-Vis spectra of PLA_Cu^o^ (**A**–**C**) and PCL_Cu^o^ (**D**–**F**) samples before and after ageing.

UV-Vis spectra were obtained for PLA and PCL compositions with copper before and after the ageing process (Figure 2). The reference PLA material exhibited an absorption band characteristic of this polymer in the UV range of approximately 230–330 nm [64]. The spectrum of PCL exhibited enhanced absorption intensity in the 230–350 nm range, indicative of carbonyl groups (C=O). Additionally, a distinctive PCL absorption band at 280 nm was observed [65]. The spectrum of the PLA_Cu^o^ and PCL_Cu^o^ samples exhibited strong broad bands within the range of 300–800 nm, which may correspond to Cu_2_O [66,67]. For commercial Cu_2_O powders (without specific morphology), a broad absorption band with a maximum in the range of 450 to 700 nm was observed, ending at approximately 900 nm [67]. Subsequent to the ageing process, a modification in the intensity of the absorbances was observed for all the spectres. Additionally, a 300–500 nm band was observed in the UV-Vis spectrum for PLA, which was likely associated with polymer degradation. The increase in the number of carboxyl groups, which have a stronger absorbance, may be the cause of changes in the transmittance of PLA during polymer photodegradation [68].

**Figure 3:**
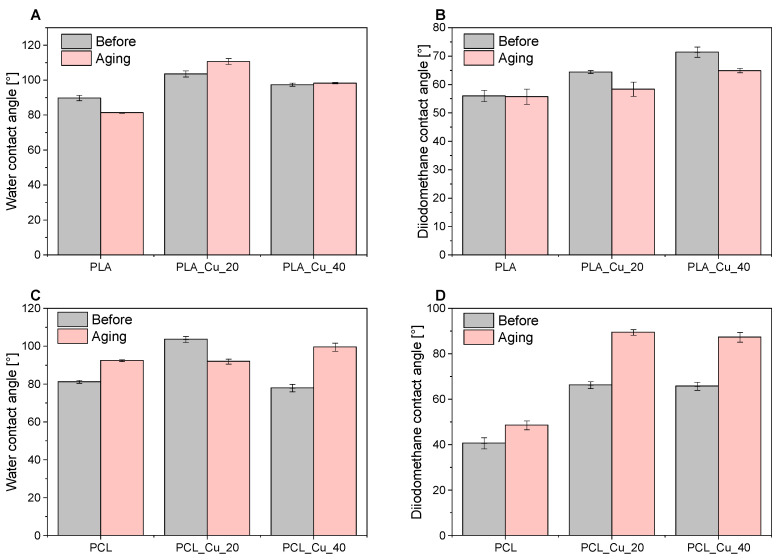
Contact angle values of PLA (**A**,**B**) and PCL (**C**,**D**) samples examined by distilled water and diiodomethane.

**Figure 4:**
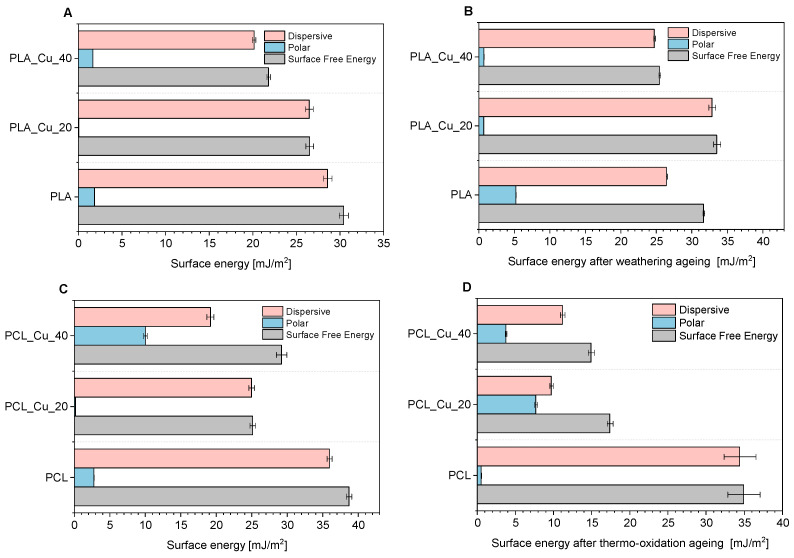
Surface free energy, polar and dispersive components of PLA (**A**,**B**) and PCL (**C**,**D**) samples before and after ageing.

Optical contact angle measurement systems are used to determine the contact angle of a surface and thus to determine its hydrophobic or hydrophilic nature. The contact angles for water and diiodomethane were measured on the surface of all samples (Figure 3). Moreover, the contact angle was used to determine surface energies, along with the polar and dispersive components, for both unaged and aged materials (Figure 4). In particular, this study focused on the effects of weathering ageing (PLA) and thermo-oxidative ageing (PCL). The contact angles for the PLA and PCL reference samples were 89.76° and 81.27° for water, and 55.99° and 40.73° for diiodomethane, respectively. It was observed that after 20 min of copper polymer spraying, the PLA and PCL samples exhibited a more hydrophobic character (wetting angles for water were 103.54° for PLA_Cu^o^_20 and 103.01° for PCL_Cu^o^_20, respectively). However, after a longer period of copper spraying, the samples regained a more hydrophilic nature (PLA_Cu^o^_40 97.42°, PCL_Cu^o^_40 77.99°). The PLA and PCL reference samples were characterised by hydrophilic properties (wetting angles for water below 90°). After 20 min of copper sputtering on the polymer matrices, their nature changed to more hydrophobic, which could have resulted from the reduction in polar interactions between the polymers and water through the copper layer. On the other hand, the longer 40-min sputtering time resulting in hydrophilic surfaces was probably related to the fact that the thin copper layer present on the polymer surface, like most metals, is hydrophilic and has a moderate contact angle [69]. Moreover, such changes in the surface properties of samples may be related to changes in their roughness after subsequent sputtering [70,71,72]. Copper was deposited on the smooth hydrophilic polymer surface (samples sputtered for 20 min), which could have increased the sample roughness and therefore reduced their hydrophilicity (the samples were more hydrophobic). However, longer copper sputtering (40 min) likely resulted in a thicker, more uniform, smoother, and less rough metal layer, contributing to more hydrophilic samples. Roughness increases the wettability (reducing the contact angle) of hydrophilic surfaces and decreases the wettability (increasing the contact angle) of hydrophobic surfaces by changing the effective solid-liquid contact area and trapping air. In summary, roughness makes a hydrophilic surface more hydrophilic and a hydrophobic surface more hydrophobic.

The polycaprolactone samples showed a decrease in surface free energy after 20 min of copper spraying by 13.58 mJ/m^2^. Prolonged exposure of the samples caused an increase in SFE, but the value remained lower than that of the reference material (29.18 mJ/m^2^). Thermo-oxidative ageing affected the PCL samples, significantly reducing these values. The surface energy results were confirmed by the wetting angle values, indicating that the thickness of the copper coating may affect the hydrophilicity of the material [73]. After magnetron sputtering of copper onto polylactide samples, the free surface energy decreased almost linearly, in contrast to polycaprolactone samples. Increasing the copper deposition time can increase surface roughness [43], thus affecting surface properties, including surface energy [50].

After thermo-oxidative ageing, PCL samples exhibited significantly lower surface energy values compared to the reference polymer. In contrast, in the case of PLA-based materials, weathering ageing caused an increase in surface energy values for both PLA and PLA_Cu^o^ samples. The differential changes in surface energy for the two materials could be associated with the conditions of thermo-oxidative and weathering ageing for individual polymers. The lower SFE values for PCL samples could be indicative of reduced oxidation of the copper layer, a consequence of the presence of oxygen and elevated temperature. Conversely, the more complex and aggressive weathering ageing conditions (humidity, elevated temperature, UV radiation) for PLA may have contributed to the degradation of the polylactide matrix and stronger oxidation of copper.

The assessment of the hydrophilicity and hydrophobicity of materials can be an important parameter in the design of products with antibacterial properties. The adhesion and subsequent growth of micro-organisms is typically contingent on the presence of a moist environment. Hydrophobic surfaces naturally repel water, thereby preventing the adhesion of microorganisms. Hydrophobicity creates a barrier that prevents water-based microorganisms, such as bacterial biofilms, from settling on the surface [74]. In the design of biodegradable materials with a magnetron-sputtered copper layer, the choice of polymer matrix and the amount of copper applied are both crucial factors in achieving the expected antibacterial properties. The most hydrophobic properties were exhibited by the PLA_Cu^o^_20 and PCL_Cu^o^_20 samples, with water contact angles of approximately 103°, suggesting that the surface properties of these samples should potentially be the most conducive to antimicrobial activity among all materials. Increased hydrophobicity is known to reduce bacterial adhesion and biofilm formation by limiting surface wettability. Surfaces with contact angles above 90° restrict moisture retention and nutrient adsorption, thereby enhancing the bacteriostatic effect [75,76]. However, in the case of the described samples, their antimicrobial properties may also be influenced by the availability and release of copper from the materials.

The antimicrobial properties of the PLA and PCL composites with magnetron-sputtered copper were evaluated in accordance with EN ISO 20645:2006 using *E. coli* (Gram-negative) and *S. aureus* (Gram-positive) as test strains.

The unmodified PLA and PCL samples showed no inhibition zones and exhibited intensive microbial growth beneath and around the samples, confirming the absence of inherent antimicrobial properties in the neat polymers. In contrast, the copper-coated materials exhibited distinct antibacterial activity. For samples with a longer sputtering time (PLA_Cu^o^_40 and PCL_Cu^o^_40), no bacterial growth was observed either beneath or around the samples, confirming a bactericidal effect. However, in the case of the PLA_Cu^o^_20 sample, partial bacterial growth under the sample was noted, indicating that a shorter copper deposition time and lower metal content may result in insufficient surface coverage to achieve full antimicrobial protection. These results are summarized in Table 4.

The obtained results confirm that the bactericidal effect strongly depends on the amount and continuity of the deposited copper layer. Thinner coatings, obtained after shorter magnetron sputtering, may not provide full coverage, enabling limited bacterial adhesion and proliferation [75,77]. These findings are consistent with our previous studies, in which the antimicrobial efficiency of copper-containing polymer composites was found to increase with higher copper loading and more uniform surface distribution of the metal [78,79]. The present work therefore supports the hypothesis that the antimicrobial performance of copper-modified biodegradable polymers results primarily from the availability of surface-exposed copper ions and their redox interaction with bacterial membranes. The observed antimicrobial behavior also correlates with the surface hydrophobicity of the materials, as higher contact angles in PLA_Cu^o^_20 and PCL_Cu^o^_20 samples limited bacterial adhesion and enhanced the bacteriostatic efficiency of the copper layer.

**Figure 5:**
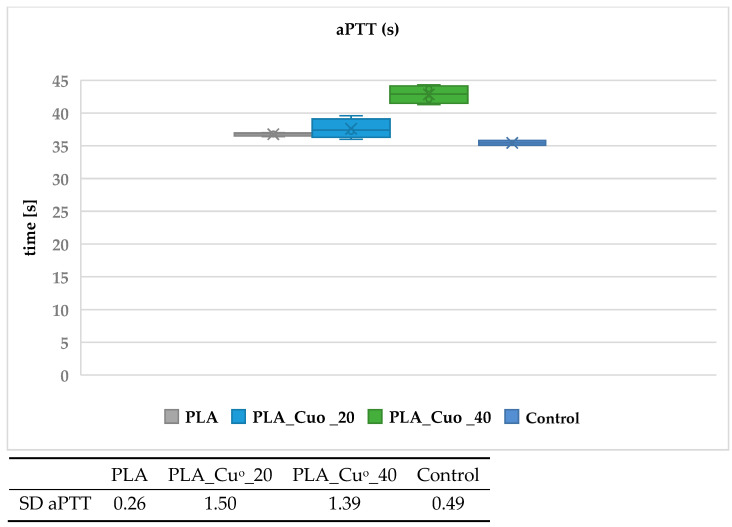
Effect of PLA compositions on activated partial thromboplastin time (aPTT). The samples: Control, plasma control; PLA; PLA_Cu^o^_20; and PLA_Cu^o^_40. The results are presented as the mean (×), median (horizontal line), range (bars), and interquartile range (box). SD stands for standard deviation.

**Figure 6:**
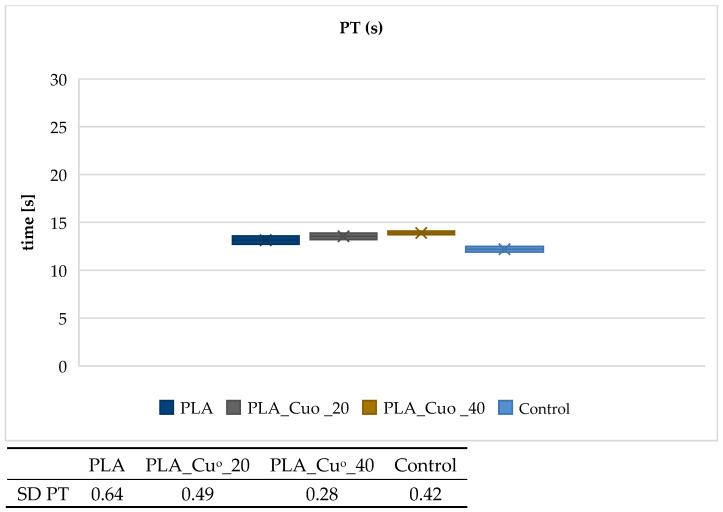
Effect of PLA compositions on pro-thrombin time (PT). The samples: Control, plasma control; PLA; PLA_Cu^o^_20; and PLA_Cu^o^_40. The results are presented as the mean (×), median (horizontal line), range (bars), and interquartile range (box). SD stands for standard deviation.

**Figure 7:**
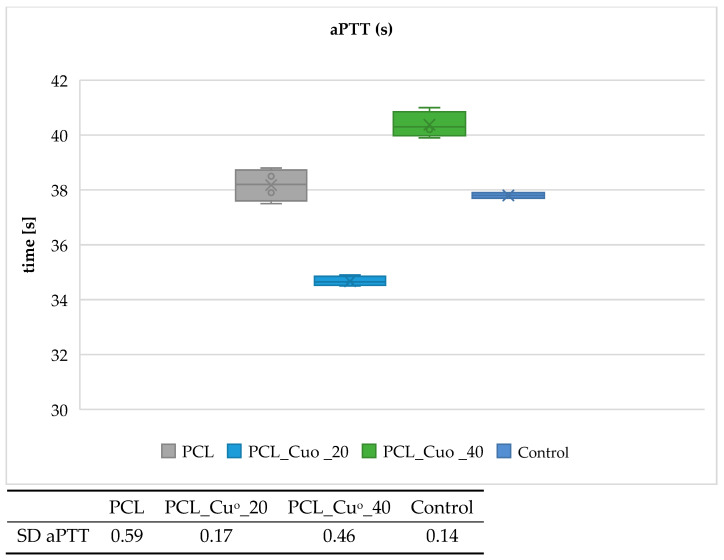
Effect of PCL compositions on activated partial thromboplastin time (aPTT). The samples: Control, plasma control; PCL; PCL_Cu^o^_20; and PCL_Cu^o^_40. The results are presented as the mean (×), median (horizontal line), range (bars), and interquartile range (box). SD stands for standard deviation.

**Figure 8:**
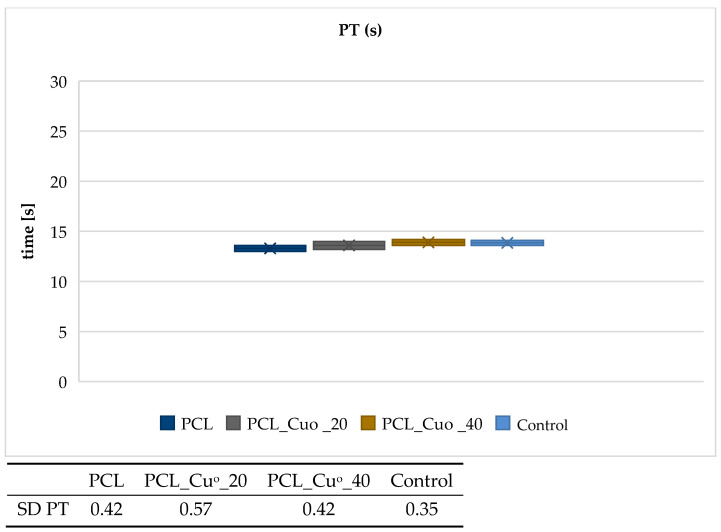
Effect of PCL compositions on activated partial thromboplastin time (aPTT). The samples: Control, plasma control; PCL; PCL_Cu^o^_20; and PCL_Cu^o^_40. The results are presented as the mean (×), median (horizontal line), range (bars), and interquartile range (box). SD stands for standard deviation.

Figure 5, Figure 6, Figure 7 and Figure 8 present the results of the activated partial thromboplastin time (aPTT) and prothrombin time (PT) analyses for the examined PLA and PCL compositions, including copper-modified and reference samples, as well as the plasma control.

The results indicate that copper deposition on biodegradable polymers influences the dynamics of blood coagulation. For both PLA- and PCL-based materials, a slight prolongation of PT and aPTT was observed in comparison with the unmodified polymers and plasma control. The effect was more pronounced for sample with higher copper loading (PLA_CuO_40), suggesting that increasing coating thickness modifies the blood–material interface and affects coagulation kinetics. The moderate extension of PT values points to a negligible impact on the extrinsic coagulation pathway, whereas the more distinct changes observed for aPTT suggest an involvement of mechanisms related to the intrinsic pathway. At this stage, however, the data do not allow a definitive attribution of this effect to copper ion–mediated processes. Rather, the observed behavior is consistent with alterations at the blood–material interface, including changes in surface chemistry and topology following copper deposition, which may influence the adsorption and organization of plasma proteins involved in contact activation. Although Cu^2+^ release was not directly quantified in the present study, the interpretation proposed here remains consistent with our earlier work on copper-modified biopolymer textiles. In studies on linen-copper composites, a reproducible modulation of coagulation parameters, including aPTT, was observed as a function of copper deposition and material architecture [79]. Comparable effects were also reported for cotton–chitosan systems incorporating copper(II) oxide and copper complexes, where the biological response depended strongly on the chemical form of copper and its interaction with the polymer matrix [80]. Moreover, investigations on wool–copper materials suggested that copper-related biological effects may arise not only from ion release but also from surface-mediated interactions and the presence of localized copper species at the biomaterial interface [81]. Taken together, these previous findings provide a coherent experimental context for the observations reported in the present work [78,79,80,81]. Nevertheless, quantitative analysis of Cu^2+^ release under assay-relevant conditions will be required to fully substantiate the proposed mechanism. Overall, the results suggest that copper-modified biodegradable composites do not induce procoagulant responses under the tested conditions. Instead, they exhibit a mild anticoagulant tendency, which may be advantageous for biomedical applications involving transient blood contact, such as bioresorbable implants or materials for transient biological contact provided that further studies confirm the underlying mechanisms and long-term hemocompatibility.

## 4. Conclusions

Polymer materials with antimicrobial properties can be produced on the basis of biodegradable polymers. Antimicrobial properties for biodegradable materials PLA and PCL have been achieved by magnetron sputtering of copper onto the surface of the materials. Polymer compositions sputtered with copper for 20 min were characterised by the most hydrophobic surface, and thus potentially the most conducive to obtaining a product with antibacterial properties; however, in the case of the described samples, the availability of copper and not hydrophobicity was the main factor influencing antimicrobial effectiveness. The Ultraviolet Protection Factor (UPF) values for the PLA and PCL samples containing copper were both greater than 50, indicating that the materials provided excellent UV protection.

The thermo-oxidative ageing of PCL and the weathering ageing of PLA resulted in changes to the surface properties of the materials. These changes included variations in the intensity of the UV-Vis spectra and the colour of the samples, which resulted from copper oxidation. There were also changes to the nature and surface energy of the compositions. Analysis of changes in the surface properties of samples after controlled ageing can be helpful in estimating the durability of copper applied to samples and can assist in estimating the potential service life of biodegradable antibacterial products.

The biological characterization confirmed that copper-modified PLA and PCL samples exhibited antibacterial activity against *E. coli* and *S. aureus*, with the exception of the PLA_Cu^o^_20 sample, where slight bacterial growth was observed due to lower copper content. The hemocompatibility studies (PT and aPTT) demonstrated that copper deposition did not trigger procoagulant responses but resulted in a mild prolongation of clotting times, indicating limited interference with the intrinsic coagulation pathway. These results suggest that the magnetron-sputtered copper layer maintains blood compatibility while imparting durable antimicrobial protection.

Overall, the presented findings highlight that biodegradable polymer–copper composites combine effective antimicrobial performance, UV stability, and hemocompatibility, making them promising candidates for advanced biomedical and packaging applications requiring both environmental safety and biological functionality.

## Figures and Tables

**Table 1:** PLA and PCL samples with copper deposited by magnetron sputtering.

Sample Name	Copper Application Time [min]	Conditions [-]
PLA	0	-
PLA_Cu^o^_20	20	1
PLA_Cu^o^_40	40	2
PCL	0	-
PCL_Cu^o^_20	20	1
PCL_Cu^o^_40	40	2

**Table 2:** Copper content applied to PLA and PCL compositions.

Sample	M Cu^o^ [g/kg]
PLA	-
PLA_Cu^o^_20	0.68
PLA_Cu^o^_40	4.00
PCL	-
PCL_Cu^o^_20	4.43
PCL_Cu^o^_40	3.55

The results have been measured in triplicate and presented as a mean value with a standard deviation of approximately 2%.

**Table 3:** Ultraviolet protection factor (UPF) values of PLA and PCL samples modified by magnetron sputtering.

Sample	UPF	UVA [%]	UVB [%]	%T [%]
PLA	4.84	50.02	14.07	41.98
PLA_Cu^o^_20	>50	0.02	0.01	0.02
PLA_Cu^o^_40	>50	0.02	0.02	0.02
PCL	7.96	26.22	8.88	22.38
PCL_Cu^o^_20	>50	0.02	0.02	0.02
PCL_Cu^o^_40	>50	0.02	0.01	0.02

The results have been measured in triplicate and presented as a mean value with a standard deviation of approximately 2%.

**Table 4:** Antibacterial activity results for PLA and PCL materials with magnetron-sputtered copper.

Sample Name	Bacterial Growth Under the Sample	*E. coli* (ATCC 25922)	*S. aureus* (ATCC 6538)
PLA (reference)	Present	+	+
PLA_Cu^o^_20	Partial	±	±
PLA_Cu^o^_40	None	-	-
PCL (reference)	Present	+	+
PCL_Cu^o^_20	None	-	-
PCL_Cu^o^_40	None	-	-

Concentration of inoculum [CFU/mL]: *E. coli* = 1.5 × 10^8^; *S. aureus* = 1.3 × 10^8^. Legend: “-” no growth; “±” slight growth beneath the sample; “+” visible bacterial growth under the sample.

## Data Availability

The original contributions presented in this study are included in the article. Further inquiries can be directed to the corresponding authors.
